# FLOWERING LOCUS T has higher protein mobility than TWIN SISTER OF FT

**DOI:** 10.1093/jxb/erv326

**Published:** 2015-07-02

**Authors:** Suhyun Jin, Hye Seung Jung, Kyung Sook Chung, Jeong Hwan Lee, Ji Hoon Ahn

**Affiliations:** Creative Research Initiatives, Department of Life Sciences, Korea University, Seoul 136–701, South Korea

**Keywords:** *Arabidopsis thaliana*, FLOWERING LOCUS T (FT), Flowering time, long-distance signalling, micrografting, TWIN SISTER OF FT (TSF).

## Abstract

In plants, successful reproduction requires the proper timing of flowering under changing environmental conditions. *Arabidopsis FLOWERING LOCUS T* (*FT*), which encodes a proposed phloem-mobile florigen, has a close homologue, *TWIN SISTER OF FT* (*TSF*). During the vegetative phase, *TSF* shows high levels of expression in the hypocotyl before *FT* induction, but the *tsf* mutation does not have an apparent flowering-time phenotype on its own under long-day conditions. This study compared the protein mobility of FT and TSF. With *TSF*-overexpressing plants as the rootstock, the flowering time of *ft tsf* scion plants was only slightly accelerated. Previous work has shown that FT is graft-transmissible; by contrast, this study did not detect movement of TSF from the roots into the shoot of the scion plants. This study used plants overexpressing FT/TSF chimeric proteins to map a region responsible for FT movement. A chimeric TSF with region II of FT (L28 to G98) expressed in the rootstock caused early flowering in *ft tsf* scion plants; movement of the chimeric protein from the rootstocks into the shoot apical region of the *ft tsf* scion plants was also detected. Misexpression of *TSF* in the leaf under the control of the *FT* promoter or grafting of *35S::TSF* cotyledons accelerated flowering of *ft-10* plants. FT was more stable than TSF. Taking these results together, we propose that protein mobility of FT is higher than that of TSF, possibly due to a protein domain that confers mobility and/or protein stability.

## Highlight

FLOWERING LOCUS T, a proposed phloem-mobile florigen, shows higher protein mobility from the rootstock to the scion than its closest homologue TWIN SISTER OF FT, possibly due to a protein domain that confers mobility and/or protein stability.

## Introduction

The ability to adjust the timing of flowering under continuously changing environmental conditions is important for successful reproduction in plants. Intricate, interconnected signalling networks have evolved to perceive inductive or repressive environmental stimuli (Srikanth and Schmid, 2011). Extensive molecular genetic analyses using *Arabidopsis thaliana* have suggested that flowering time is controlled by multiple, interdependent genetic pathways, including the photoperiod, autonomous, vernalization, gibberellic acid, and ambient temperature pathways (Amasino and Michaels, 2010). Under long-day (LD) conditions, genes that act in the photoperiod pathway play a major role in controlling flowering.

*FLOWERING LOCUS T* (*FT*) is an important regulator of flowering time in *Arabidopsis* (Kardailsky *et al.*, 1999; Kobayashi *et al.*, 1999). The *A. thaliana* genome has five additional genes homologous to *FT*, namely, *TERMINAL FLOWER 1* (Shannon and Meeks-Wagner, 1991), *TWIN SISTER OF FT* (*TSF*) (Michaels *et al.*, 2005; Yamaguchi *et al.*, 2005), *MOTHER OF FT AND TFL1* (Yoo *et al.*, 2004), *BROTHER OF FT AND TFL1* (Yoo *et al.*, 2010), and *ARABIDOPSIS THALIANA CENTRORADIALIS HOMOLOGUE* (Mimida *et al.*, 2001). Although all of these genes affect flowering time, *FT* receives particular attention because FT protein may function as the long-sought florigen or, at least, as an important component of the florigen pathway (Zeevaart, 2008). This notion is supported by studies showing that FT is remotely regulated from the leaf vein (Takada and Goto, 2003), shows a molecular/genetic interaction with the transcription factor FD in the shoot apical meristem (SAM) (Abe *et al.*, 2005; Wigge *et al.*, 2005), and can be transmitted across the graft junction (Corbesier *et al.*, 2007). The detection of FT in the phloem sap (Giavalisco *et al.*, 2006) provided support for the idea that FT constitutes an important part of a mobile florigen.

*TSF* is the closest homologue of *FT* in *Arabidopsis* (82% identical amino acids). As with *FT*, *TSF*-overexpressing plants flower extremely early (Yamaguchi *et al.*, 2005). However, the effect of *tsf* loss of function is very weak or almost undetectable under LD conditions. In contrast, under short-day (SD) conditions, where the *ft* mutation shows a very limited effect, the *tsf* mutation causes a strong late-flowering phenotype (Yamaguchi *et al.*, 2005) and *TSF* expression in the leaf gradually increases in an age-dependent manner (Hiraoka *et al.*, 2013). Recent work has shown that *TSF* plays a role in flowering promoted by cytokinin under non-inductive SD conditions (D’Aloia *et al.*, 2011). These results suggested that *FT* and *TSF* play overlapping roles in the promotion of flowering under LD conditions and are differentially regulated by different floral-inducing signals (D’Aloia *et al.*, 2011).

A number of reports have suggested that both *FT* and *TSF* are regulated in a similar fashion. Transcription of *FT* and *TSF* is activated by CONSTANS (CO) (Samach *et al.*, 2000; Suárez-López *et al.*, 2001; Yamaguchi *et al.*, 2005), a B-box zinc finger transcription factor that plays an important role in the photoperiod pathway, and is repressed by a complex containing SHORT VEGETATIVE PHASE (SVP) and FLOWERING LOCUS M (FLM) (Lee *et al.*, 2013; Pose *et al.*, 2013). Interestingly, expression of *CO*, *TSF*, and *FT* is detected in the vasculature (probably in phloem companion cells) (Abe *et al.*, 2005; Takada and Goto, 2003; Yamaguchi *et al.*, 2005), further supporting the hypothesis that CO may regulate both *FT* and *TSF* cell-autonomously in the phloem to trigger flowering. Also, PKDM7B (also known as JUMONJI14) mediates H3K4 demethylation of both *FT* and *TSF* (Yang *et al.*, 2010). In addition to the co-regulation of *FT* and *TSF*, other evidence suggests that TSF remotely regulates flowering from its main expression domain, as FT also does. For example, TSF interacts with FD (Jang *et al.*, 2009), phloem-specific *TSF* misexpression driven by the *SUC2* promoter induces early flowering (Jang *et al.*, 2009), and TSF is present in in phloem sap (Giavalisco *et al.*, 2006).

Despite their similarities, some differences in *FT* and *TSF* expression and mutant phenotypes suggest that their functions may also differ. For example, high expression of *TSF* occurs in the hypocotyl and the basal part of the cotyledon before *FT* induction in response to photoperiod (Yamaguchi *et al.*, 2005). At later stages, *TSF* is also expressed in the veins of mature leaves. However, not much is known about why the *tsf* mutation has only a weak effect under LD conditions. The present study tested the movement of TSF by using micrografting surgeries in *Arabidopsis*. TSF showed low protein mobility, in sharp contrast to FT. Domain-swapping experiments identified a region of FT that conferred TSF movement from rootstock to scion. In addition, FT was more stable than TSF. These results suggest that FT has a greater ability to move than TSF.

## Materials and methods

### Plant materials and growth conditions

All of the mutants used in this study were in the *A. thaliana* Columbia (Col) background. The *ft-10* and *tsf-1* mutants are T-DNA insertion mutants described elsewhere (Yamaguchi *et al.*, 2005; Yoo *et al.*, 2005). The plants were grown in soil or Murashige–Skoog (MS) medium (half-strength) at 23 °C under LD conditions (16h light/8h dark cycle) at a light intensity of 70 μmol m^–2^ s^–1^. The flowering times of the plants are expressed as the total number of rosette and cauline leaves at flowering.

### Plasmid construction

To generate *pFT::HA:FT:T7* and *pFT::HA:TSF:T7* constructs, first, the 35S promoter present in the pCHF3 vector (Neff *et al.*, 1999) was substituted with the 8.1kb *FT* promoter, which contains sufficient regulatory information to mimic wild-type *FT* gene expression (Adrian *et al.*, 2010). The *FT* or *TSF* coding sequence was fused with an HA tag at the N-terminus and with a T7 tag at the C-terminus. The resulting chimeric sequence was cloned into the modified pCHF3 vector by using the In-Fusion HD Kit (Clontech, USA). The recombinant plasmid was transformed into *Agrobacterium* strain GV3101 and introduced into *ft-10* plants by floral dip infiltration (Clough and Bent, 1998). To generate *35S::FT:T7* and *35S::TSF:T7* constructs, the *FT* or *TSF* coding sequence was fused with a T7 tag at the C-terminus and the resulting chimeric sequence was cloned into the pCHF3 vector containing the 35S promoter. The *pTSF::FT* construct was generated by fusing the 2.1kb *TSF* promoter amplified using JH6610 and JH6611 primers with the *FT* coding sequence. The primers used in this study are described in Supplementary Table S1 (available at *JXB* online).

### Micrografting

Five-day-old seedlings grown under LD conditions at 23 °C were subjected to micrografting surgeries. A recipient scion was grafted on to the hypocotyl of a donor rootstock in butt-grafting experiments (Turnbull *et al.*, 2002). Cotyledon grafting experiments were performed as described previously (Yoo *et al.*, 2013*b*). The resulting grafts were kept at 25 °C for 1 week under continuous light conditions on MS plates. The surviving grafts were planted on soil and grown under LD conditions at 23 °C.

### Generation of *FT/TSF* chimeric constructs

To generate *Arabidopsis* transgenic plants overexpressing *FT/TSF* chimeras, six chimeric sequences were designed and synthesized (Bioneer, Korea). The chimeric sequences were fused with a T7 tag at their C-terminus. The resulting constructs were cloned into the pCHF3 vector containing the 35S promoter. The recombinant plasmids were introduced into wild-type *Arabidopsis* using the floral dip method (Weigel and Glazebrook, 2002). Transgenic plants were selected for kanamycin resistance. Lines carrying a single T-DNA locus were selected on the basis of their segregation ratio. Protein expression levels of chimeric proteins from each line were measured. A homozygous plant was selected in the T3 generation and used for subsequent studies.

### Immunoprecipitation

Protein extracts were prepared from the shoot apical region of *ft tsf* scion plants grafted to plants overexpressing the various FT/TSF chimeric proteins. After pre-cleaning of the total protein extracts with protein A/G plus agarose (Santa Cruz Biotechnology, USA), the soluble extracts were mixed with anti-T7 monoclonal antibody (Novagen, USA) and incubated overnight. To precipitate immunocomplexes, 20 µl of protein A/G plus agarose beads was added and further incubated, with gentle agitation. To remove unbound proteins, the beads were washed three times in washing buffer (extraction buffer without β-mercaptoethanol). Immunoprecipitated proteins were analysed by western blotting using anti-T7 polyclonal antibody (Thermo Scientific, USA).

### Expression analysis

For quantitative PCR (qPCR), total RNA was isolated using TRIzol reagent (Invitrogen, USA), according to the manufacturer’s instructions. A 1 µg aliquot of total RNA was treated with DNaseI (New England Biolabs, USA) and used for cDNA synthesis with the First-Strand Synthesis Kit (Roche Applied Science, USA). Transcript levels were analysed by qPCR as described previously (Hong *et al.*, 2010; Udvardi *et al.*, 2008). The qPCR analysis was carried out in 384-well plates with a LightCycler 480 (Roche Applied Science, USA) using KAPA SYBR Green Master mixture and Roche master mixture. The following program was used for amplification: pre-denaturation for 5min at 94 °C, followed by 40 cycles of denaturation for 10 s at 94 °C, annealing for 10 s at 60 °C and elongation for 10 s at 72 °C. The primers used in this study are described in Supplementary Table S1.

For western blot analysis, crude protein extracts were prepared in PRO-PREP buffer (Invitrogen, USA). The proteins were separated by 15% SDS-PAGE and blotted on to a polyvinylidene difluoride membrane. The membrane was blocked in 5% skimmed milk and TBST [10mM Tris, 150mM NaCl, and 0.1% Tween-20 (pH 7.5)] buffer. Primary antibody (anti-T7 antibody) was diluted to 1:3000 and incubated overnight at 4 °C. A subsequent incubation with secondary antibody [anti-rabbit IgG horseradish peroxidase (Santa Cruz Biotechnology, diluted to 1:5000) or anti-mouse IgG horseradish peroxidase (Sigma Aldrich, diluted to 1:5000)] followed. The membrane was visualized by chemiluminescence using ECL detection reagent (RPN2235; GE Healthcare).

### Protein stability test

*35S::FT:T7* and *35S::TSF:T7* seedlings were grown on MS plates until 7 days after germination. They were transferred to liquid MS medium supplemented with 500 µM cycloheximide (CHX) (Liu and Stone, 2010). Vacuum was applied for 30min. Whole seedlings were harvested at 6, 9, 12, and 18h. Approximately 5 µg of total protein isolated from *35S::FT:T7* and *35S::TSF:T7* seedlings was separated on 15% SDS-PAGE and blotted with anti-T7 monoclonal antibody (Novagen, USA).

## Results

### Micrografting the hypocotyl from wild-type plants and a *TSF*-overexpressing line did not substantially accelerate flowering of *ft tsf* scion plants

**Fig. 1.**
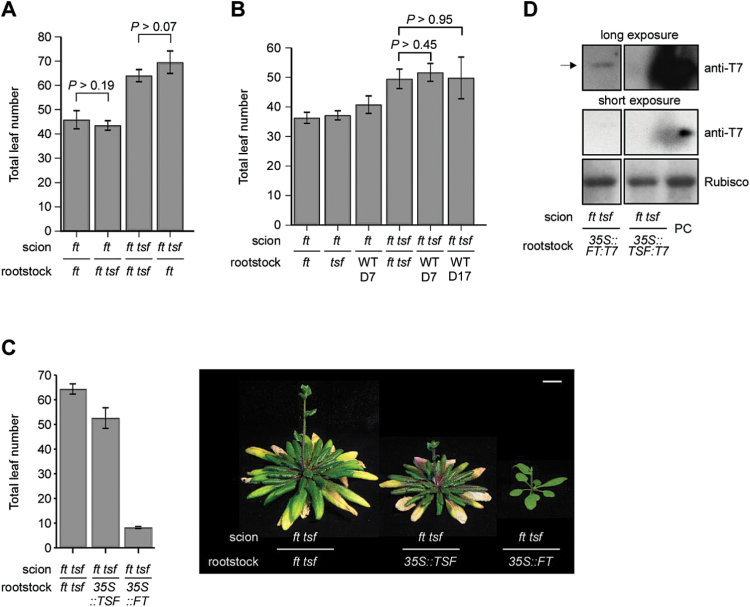
Effect of grafting wild-type (WT) and *TSF*-expressing rootstocks on the flowering time of scion plants under LD conditions. (A) Flowering time of butt-grafted plants. Five-day-old scion and rootstock plants were grafted. Student’s *t*-test analysis indicated that neither the difference in flowering time between *ft*/*ft* and *ft*/*ft tsf* plants nor the difference in flowering time between *ft tsf*/*ft tsf* and *ft tsf*/*ft* plants was statistically significant. (B) Effect of age of wild-type rootstock plants on flowering time of *ft tsf* scions. Student’s *t*-test analysis indicated that the flowering time of *ft tsf*/WT Col (D7) and *ft tsf*/WT Col (D17) plants did not significantly differ from that of *ft tsf*/*ft tsf* grafted plants. Note that the grafted plants in this experiment flowered earlier than in other experiments due to different growth conditions with stronger light intensity. D: day. (C) Strong acceleration of flowering of the *ft tsf* scion by overexpression of *FT* and a weak effect of overexpression of *TSF*. Scale bar = 1cm. (D) Western blot analysis showing the absence of TSF:T7 protein in the shoot apex of *ft tsf* scion plants grafted to *35S::TSF:T7* plants. In contrast, a band (arrow) was detected in protein extracts from the shoot apex of *ft tsf* scion plants grafted to *35S::FT:T7* plants. A 5 µg aliquot of protein extract prepared from *35S::FT:T7* plants was used as a positive control (PC). (This figure is available in colour at *JXB* online.)

To test whether the high expression of *TSF* in the hypocotyl during the vegetative phase (Supplementary Fig. S1) regulated flowering, it was first examined whether butt-grafting a wild-type hypocotyl could alter flowering time. Either *ft* single mutants or *ft tsf* double mutants were used as the rootstock and *ft tsf* double mutants were used as the scion. To exclude the possibility of *TSF* expressed in the hypocotyl of the scion affecting the result, the hypocotyl was removed from the scion (Supplementary Fig. S2). If TSF in the hypocotyl of the rootstock is graft-transmissible and can regulate flowering, the flowering of *ft tsf* scion plants grafted to a *ft* rootstock (a grafting combination hereafter described as *ft tsf*/*ft*, scion/rootstock) should be accelerated, albeit slightly, as the *ft* rootstock plants carried an endogenous wild-type *TSF* gene. However, the *ft tsf*/*ft* and *ft tsf*/*ft tsf* plants flowered with similar numbers of leaves (69.5 versus 64.0 leaves; *P*>0.07) (Fig. 1A and Supplementary Fig. S3). Similarly, using *ft tsf* double mutants as a rootstock did not delay the flowering time of *ft* scion plants. *ft*/*ft* and *ft*/*ft tsf* plants flowered with similar numbers of leaves (45.8 versus 43.5 leaves; *P*>0.19).

Since *TSF* expression in the hypocotyl increased in older seedlings (Supplementary Fig. S1) (Yamaguchi *et al.*, 2005), it was then tested whether grafting to the hypocotyl of older seedlings caused a visible effect. Flowering times of *ft tsf* scion plants grafted to 7- or 17-day-old wild-type rootstock plants were compared. The *ft tsf* scion plants grafted to young or mature wild-type rootstocks flowered with very similar numbers of leaves (51.7 and 49.8 leaves, respectively), which were also similar to the flowering time of *ft tsf*/*ft tsf* control plants (49.5 leaves) (Fig. 1B). This analysis suggested that the higher level of *TSF* expression in the hypocotyl did not alter the flowering of scion plants. Furthermore, butt-grafting *ft*, *tsf*, or wild-type plants to *ft* mutants did not change the flowering time of *ft* scion plants (Fig. 1B). These results suggested that the absence or presence of endogenous *TSF* and/or *FT* function in rootstock plants did not alter the flowering time of micrografted scion plants.

Since endogenous levels of *TSF* did not affect the flowering of the scion, *TSF*-overexpressing plants were used for the grafting experiment. *35S::TSF* plants (Yamaguchi *et al.*, 2005) were used as a rootstock and butt-grafted to *ft tsf* double mutants. Interestingly, grafting *35S::TSF* plants to *ft tsf* mutants caused weak acceleration of flowering (Fig. 1C). The *ft tsf*/*35S::TSF* plants flowered with 52.6 leaves under LD conditions, in comparison to *ft tsf*/*ft tsf* plants, which flowered with 64.3 leaves. By contrast, grafting using *35S::FT* plants caused dramatic acceleration of flowering in *ft tsf* scion plants. The *ft tsf*/*35S::FT* plants had 8.2 leaves at flowering, close to the flowering time of *35S::FT* plants, which had 4–5 leaves at flowering (Kardailsky *et al.*, 1999; Kobayashi *et al.*, 1999).

To exclude the possibility that the weak effect on flowering was due to weak *TSF* expression in the *35S::TSF* plants that were used, *TSF* transcript levels in the rootstock plants were measured. qPCR analysis confirmed the strong expression of *TSF* (approximately 2000-fold more than in wild-type plants) in the *35S::TSF* rootstock plants that were used (Supplementary Fig. S4A). Furthermore, the ungrafted *35S::TSF* plants flowered extremely early under LD conditions (Supplementary Fig. S4B) which was indistinguishable from the phenotype of *35S::FT* plants (Kobayashi *et al.*, 1999; Yamaguchi *et al.*, 2005). These analyses revealed that although used *TSF*-overexpressing plants that showed very strong early flowering were used as rootstock, grafted scion plants did not show a substantial change in flowering time, in sharp contrast to the dramatic acceleration in flowering time observed in scion plants grafted to *35S::FT* plants.

The weak effect of grafting *35S::TSF* plants in accelerating flowering raised two possibilities: (i) TSF does not move to the *ft tsf* scion, and (ii) TSF moves to the *ft tsf* scion but fails to induce flowering at the shoot apex. To determine whether TSF moved in the butt-grafted plants, western blot analysis was performed using protein extracts prepared from the shoot apical region of *ft tsf* scion plants, and blotting an excess (400 µg) of protein. A band (arrow in Fig. 1D) was detected from the shoot apex of *ft tsf* scion plants grafted to *35S::FT:T7* plants; however, such a band was absent from the shoot apex of *ft tsf* scion plants grafted to *35S::TSF:T7* plants. This result suggested that FT:T7 protein moved to the shoot apex of the scion plants, but TSF:T7 protein movement was almost completely absent. Expression levels of FT:T7 and TSF:T7 proteins were similar in the rootstock of surviving grafts (Supplementary Fig. S5), excluding the possibility that absent or low expression of TSF:T7 in the rootstock was the cause of the failure to detect TSF:T7 in the scion plants. Taken together, these analyses revealed that *FT* overexpression had a strong effect on flowering of grafted scions, whereas *TSF* overexpression had little effect.

### Generation of *FT/TSF* chimeras

To map the regions responsible for the different effects of *FT* and *TSF* on accelerating flowering in grafted plants, transgenes were constructed that encoded chimeric proteins with three regions of FT and TSF swapped. *FT* and *TSF* encode proteins with the same number of amino acids (175 residues) and have only 32 substitutions, without insertions/deletions. Three regions were selected on the basis of their relative amino acid sequence similarities (Supplementary Fig. S6A): Region I showed a 37% difference in amino acid residues between FT and TSF, Region II showed only an 8% difference between FT and TSF, and Region III showed a 20% difference between FT and TSF (Supplementary Fig. S6B). Here, the chimeras are described by use of a notation that indicates the origin of each region; for instance, *FTF* encodes a chimeric protein with Regions I and III from *FT*, and Region II from *TSF*. All chimeric constructs were fused with a T7 tag and expressed under the control of the 35S promoter in wild-type plants.

**Fig. 2.**
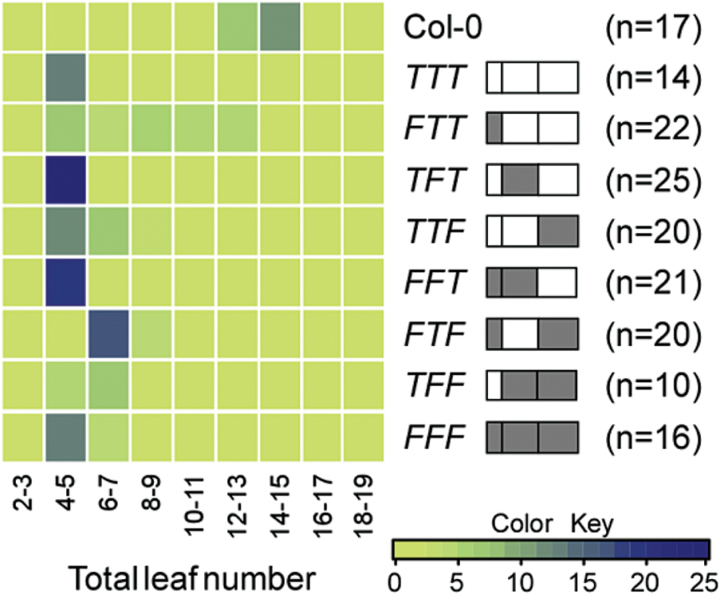
Flowering times of homozygous transgenic plants overexpressing FT/TSF chimeric proteins. Distribution of flowering time of *FT/TSF* chimeras under LD conditions, presented as a heat map. The structure of each chimeric gene is shown next to the name of each construct. Sequences of *FT* and *TSF* are shown as grey and open boxes, respectively. n = number of plants measured. *F*: region originating from *FT*; *T*: region originating from *TSF*.

The flowering time of homozygous plants from each line was measured under LD conditions. Most chimeras showed strong acceleration of flowering under LD conditions, similar to that seen in *FT*- and *TSF*-overexpressing plants (Fig. 2). *TFF, FFT, TTF*, and *TFT* were as effective at accelerating flowering as authentic *FT* or *TSF*. However, the *FTT* chimera was slightly less effective. Notably, none of the lines showed delayed flowering or unaltered flowering, suggesting that cosuppression or instability of chimeric proteins did not occur. These results indicated that these chimeras were functional in transgenic plants and acted in a similar way to parental *FT* and *TSF*.

### Flowering time of *ft tsf* scion plants micrografted to *FT/TSF* chimeras

**Fig. 3.**
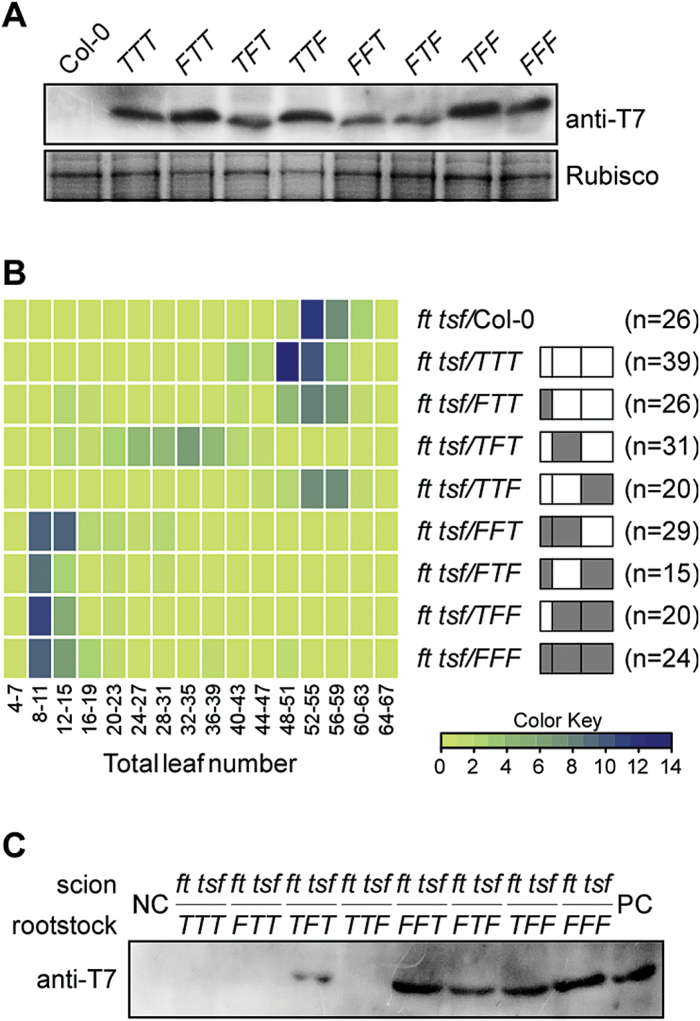
Flowering times of *ft tsf* scion plants butt-grafted to plants overexpressing FT/TSF chimeric proteins. (A) Expression of FT/TSF chimeric proteins in the rootstock used for butt-grafting. *F*: region originating from *FT*; *T*: region originating from *TSF*. (B) Distribution of flowering time of grafted *ft tsf* scion plants under LD conditions, presented as a heat map. The structure of each chimeric gene expressed in the plants used for rootstock is shown next to the name of each construct. Sequences of *FT* and *TSF* are shown as grey and open boxes, respectively. n = number of plants measured. (C) Detection of FT/TSF chimeric proteins in the shoot apical region of *ft tsf* scion plants. Extracted proteins were immunoprecipitated with anti-T7 monoclonal antibody and immunoblotted anti-T7 polyclonal antibody. Protein extracts from the non-grafted *35S::FT:T7* seedlings and from wild-type Col-0 plants were used as a positive control (PC) and a negative control (NC), respectively.

Butt-grafting was performed to examine the effect of grafting rootstocks of homozygous plants expressing *FT/TSF* chimeras to *ft tsf* scion plants. First, the expression of FT/TSF chimeric proteins in the rootstock was confirmed (Fig. 3A). A striking change was observed in the flowering time of the scion plants grafted to rootstocks overexpressing *TFT*, in which Region II of *TSF* was substituted for Region II of *FT* (Fig. 3B). Many of the *ft tsf/TFT* lines showed flowering times that were intermediate between those of *ft tsf/FFF* and *ft tsf/TTT* lines, suggesting that TFT protein may move to the shoot apex to induce early flowering. However, grafting to plants expressing *TTF* and *FTT* still resulted in late flowering in the *ft tsf* scion, similar to that seen with *TTT*, indicating that substitution of Region I and III did not affect flowering time. It should be noted that since overexpression of *FTT* was slightly less effective than overexpression of other chimeric genes (Fig. 2), we cannot exclude the possibility that FTT protein is partially functional and this may contribute to the failure to accelerate the flowering time of scion plants. Interestingly, grafting of *FFT*, *FTF*, and *TFF* resulted in early flowering, similar to that seen for *FFF*. In particular, grafting of plants expressing *FTF,* the opposite construct of *TFT*, did not cause a delay in flowering time. This indicated that substitution of any single region of *FT* with *TSF* failed to change flowering time. In addition, consistent flowering time changes from *ft tsf* scion plants grafted to independent *FTF*- and *TFT*-overexpressing lines were observed (Supplementary Fig. S7).

Next, it was examined whether chimeric proteins moved from the rootstock to the scion. To increase the sensitivity of detection, the total protein extracts isolated from the shoot apical region of the *ft tsf* scion were immunoprecipitated using anti-T7 antibody, and western blot analysis was performed. A band was detected from *ft tsf* scion plants grafted to *TFT-, FFT-, FTF-, TFF*-, and *FFF*-overexpressing rootstocks (Fig. 3C), suggesting that the acceleration of flowering time of the *ft tsf* scion grafted to these lines is likely to be due to movement of the chimeric protein to the scion. No band was detected in *ft tsf* scions grafted to *TTT*-, *FTT*, and *TTF*-overexpressing rootstock, which was consistent with their unaltered flowering time (Fig. 3B). These results suggested that movement of chimeric protein from the rootstock caused alteration of flowering time in the scion, and further indicated that Region II of FT, which differs from TSF by only six amino acid residues, is an important domain in conferring mobility to TSF.

### Acceleration of flowering by expression of *TSF* under the control of the *FT* promoter or by grafting of a *35S::TSF* cotyledon

**Fig. 4.**
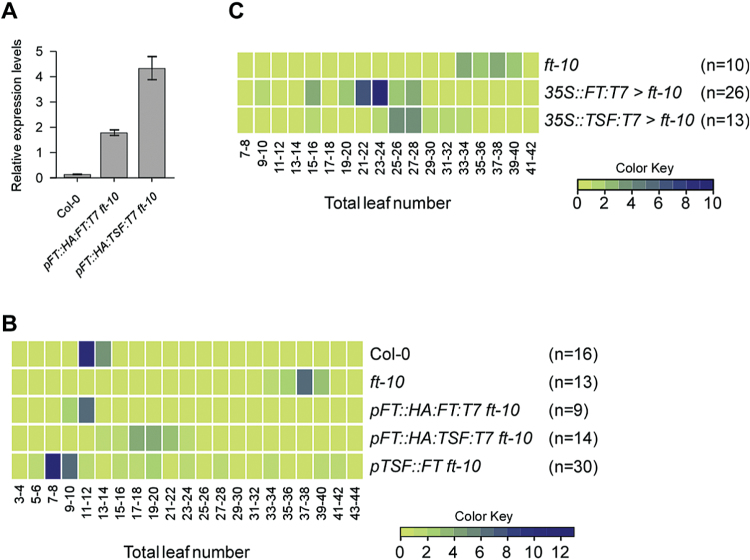
Partial rescue of the late-flowering phenotype of *ft-10* mutants by *TSF* misexpression from the *FT* promoter or by grafting of a *35S::TSF* cotyledon. (A) RNA levels of the *FT* promoter-driven *HA:FT:T7* and *HA:TSF:T7*. Total RNA was extracted from 10-day-old whole seedlings. (B) Distribution of flowering time of *pFT::HA:TSF:T7 ft-10, pFT::HA:FT:T7 ft-10*, and *pTSF::FT ft-10* plants under LD conditions, presented as a heat map. Note that *pFT::HA:TSF:T7* shows a weak effect in rescuing the late flowering of *ft-10* mutants. n = number of plants measured. (C) Distribution of flowering time of *ft-10* recipients grafted to a cotyledon of *FT*- or *TSF*-overexpressing lines. Note that flowering in *ft-10* recipient mutants grafted to a cotyledon of *35S::TSF:T7* plants was accelerated but its effect was weaker than that of a *35S::FT:T7* cotyledon. n = number of plants measured.

Because *TSF* expression in the hypocotyl or rootstock failed to accelerate flowering, we next investigated whether *TSF* expression under the control of the *FT* promoter (Adrian *et al.*, 2010) could accelerate flowering. To this end, the flowering time of *pFT::HA:FT:T7 ft-10* and *pFT::HA:TSF:T7 ft-10* plants was measured. qPCR was used to confirm the expression of the *FT* promoter-driven *FT* and *TSF* in homozygous lines (Fig. 4A). Flowering time measurement revealed that the *pFT::HA:FT:T7 ft-10* plants flowered with 11.2 leaves, indicating that *pFT::HA:FT:T7* almost completely rescued the late flowering of *ft-10* mutants (wild-type plants = 12.1 leaves and *ft-10* plants = 37.4 leaves) (Fig. 4B). By contrast, homozygous *pFT::HA:TSF:T7 ft-10* plants were slightly later flowering (18.9 leaves; *P*<0.0005) than *pFT::HA:FT:T7 ft-10* plants. We also tested whether the *TSF* promoter-driven *FT* affected the flowering time of *ft-10* mutants. *pTSF::FT ft-10* plants showed acceleration of flowering (13.9 leaves).

Further, we tested whether FT and TSF in the leaf had different abilities to rescue the phenotype of *ft-10* mutants. To this end, cotyledon micrografting experiments were performed using *35S::FT:T7* and *35S::TSF:T7* cotyledons (Yoo *et al.*, 2013*b*). The *35S::FT:T7*>*ft-10* plants flowered with 21.9±4.2 leaves (Fig. 4C), whereas *35S::TSF:T7*>*ft-10* plants flowered with 26.3±4.2 leaves (in comparison, *ft-10* mutants flowered with 36.3±2.1 leaves.) These results suggested that grafting a *35S::FT* cotyledon led to a stronger acceleration of flowering than grafting a *35S::TSF* cotyledon (*P*<0.005), and that *TSF* expressed in the leaf could function in a similar way to *FT*, albeit more weakly.

### FT protein is more stable than TSF protein

**Fig. 5.**
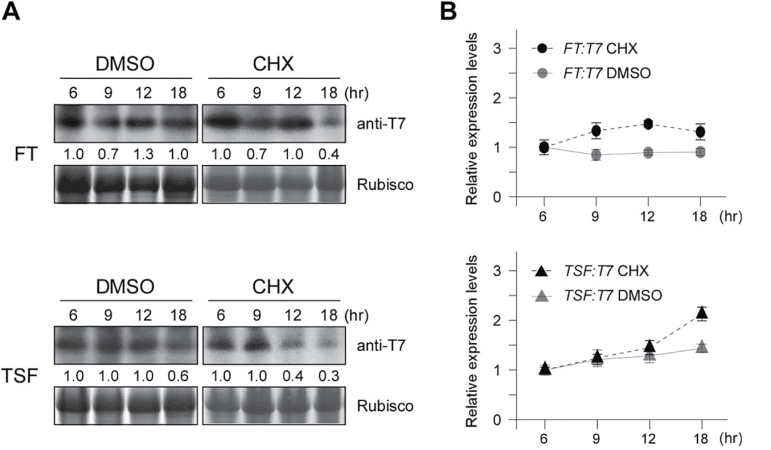
FT is more stable than TSF. (A) FT and TSF levels at each time point determined by western blot analysis using anti-T7 antibody (top). Rubisco was used as a loading control (bottom). The numbers below each band denote fold-change relative to the FT or TSF levels at 6h. CHX: cycloheximide; DMSO: dimethyl sulphoxide, the non-CHX control. (B) Relative expression levels of *FT* and *TSF* as measured by qPCR. Expression levels of *TSF* or *FT* at 6h were set to 1.

Because protein stability is important during the overall process of FT and TSF protein movement, namely, before phloem loading, during transport, and even after delivery to the SAM, the observed difference in flowering induced by FT and TSF may be associated with their different protein stabilities. To test this hypothesis, 7-day-old *35S::TSF:T7* and *35S::FT:T7* seedlings grown on MS media were treated with the protein synthesis inhibitor CHX and FT and TSF levels were analysed at the time points indicated in Fig. 5. Although both FT and TSF levels decreased following CHX treatment, TSF levels decreased more rapidly. TSF levels decreased by approximately 60% after 12h, whereas FT levels decreased by approximately 60% after 18h (Fig. 5A), indicating that FT had a longer half-life. Measurement of *TSF:T7* and *FT:T7* mRNAs after CHX treatment showed that *FT:T7* and *TSF:T7* mRNAs were consistently expressed after CHX treatment (Fig. 5B), excluding the possibility that the different decreases in protein levels might result from different decreases in their mRNA levels. These results suggested that the stronger effect of *pFT::HA:FT:T7* and *35S::FT:T7* cotyledons in rescuing the flowering time of plants bearing the *ft-10* mutation resulted from the higher stability of FT compared with TSF.

## Discussion

This study compared the protein mobility of FT and TSF using micrografting and found that FT has a greater ability to move than does TSF. The study also found that Region II of FT can confer mobility on TSF.

Although *TSF* is the closest homologue of *FT* in *Arabidopsis* (Kim *et al.*, 2013; Yamaguchi *et al.*, 2005), the function of TSF as a mobile inducer of flowering awaits experimental confirmation (Turck *et al.*, 2008). The findings of the present study suggest that FT has higher protein mobility than TSF, based on the following findings. First, micrografting experiments using wild-type and *TSF*-overexpressing plants only weakly affected the flowering time of scion plants (Fig. 1). Second, we failed to detect TSF:T7 protein in the shoot apex of scion plants grafted to *35S::TSF:T7* plants, whereas FT:T7 protein produced in rootstock plants can be detected in the shoot apex of scion plants (Notaguchi *et al.*, 2008) (Fig. 1D). Some experiments have detected transmission of a tag alone across the graft junction, for instance, green fluorescent protein (Itaya *et al.*, 2000); however, the present study failed to detect TSF:T7 protein in the shoot apex of the scion plants, suggesting that TSF has a low ability to move. Although TSF movement from the rootstock was not observed, we cannot exclude the possibility that the micrografts using *35S::TSF* plants failed to establish a functional vascular reconnection. However, this effect would have had to occur specifically in *35S::TSF* plants, as the other experiments showed transmission of FT and FT/TSF chimeric proteins.

The domain-swapping experiments showed that Region II of FT can confer mobility on TSF (Fig. 3B). The flowering time of the scion was consistent with the detection of chimeric proteins in the shoot apical region of the *ft tsf* scion. However, the rescue of the late flowering of the *ft tsf* scion by grafting could be affected not only by the mobility of the chimeric proteins, but also by the levels of expression proteins produced in the rootstock. Thus, we cannot exclude the possibility that a chimeric protein that has low mobility but high levels of expression may give a similar result to that of a chimeric protein that that has high mobility but low levels of expression. It is notable that FTF protein, in which Region II of FT is replaced with Region II of TSF, retains the ability to move to the scion plants. Thus, it seems likely that Regions I and III of FT contain key residues that are important for conferring protein mobility to TSF. The sequences of Region II of FT and TSF differ by only six residues (three non-conserved changes: Q49L, R53I, L82Q, and three conserved changes: Q34H, E59D, L61F). One of these substitutions or combinations may thus be responsible for the observed difference in protein mobility of FT and TSF. The substitution could cause a conformational change or cause dysfunction of the protein molecules, which may affect FT/ TSF–target interaction. Consistent with this notion, a conservative substitution (E to D) in an E3 ubiquitin ligase, UBR1, inhibits substrate binding due to the difference in the length of amino acid residues (Choi *et al.*, 2010). In addition, recent work using a mutational approach identified several residues important for FT function and FT movement (Yoo *et al.*, 2013*a*). Interestingly, this study identified three mutations that render FT movement-defective (V70, S76, and R83), all three of which occur in Region II, suggesting an important role of Region II. It will thus be interesting to identify the important residue(s) in Region II that confer(s) long-distance mobility to TSF.

In relation to the question of what might explain the behaviour of TSF in the control of flowering, several lines of evidence can be considered. First, protein mobility of TSF from the hypocotyl and the rootstock is low (Fig. 1), although *TSF* is mainly expressed in the hypocotyl during the vegetative phase (Yamaguchi *et al.*, 2005) (Supplementary Fig. S1). Second, *TSF* is expressed in the leaf vein in mature plants (Yamaguchi *et al.*, 2005) (Supplementary Fig. S1). Third, TSF accelerates flowering when produced under the control of the *FT* promoter, which is expressed at earlier stages in the leaf vein, where *TSF* is expressed in mature plants (Fig. 4). Fourth, grafting of a *35S::TSF* cotyledon accelerates flowering. These results suggest the possibility that although TSF produced in the hypocotyl does not move, TSF produced in the leaf can move. However, the possibility cannot be excluded that the small hypocotyl may not produce enough protein for highly effective floral induction, whereas the more extended phloem of the leaf system of older plants may produce sufficient protein to be effective. On the basis of the findings of this study, we propose that the later induction of TSF in the leaf vein of mature plants is important for floral induction and acts as a ‘fail-safe’ mechanism to ensure successful flowering even when *FT* function is defective. When *FT* is functional, FT produced in the embryonic leaf in young seedlings moves to the shoot apex and is sufficient to induce flowering (Yoo *et al.*, 2013*b*). In this case, TSF produced in the leaf vein of mature plants does not contribute to floral induction. However, when *FT* is defective or non-functional, TSF is produced in the leaf vein of mature plants and probably moves to the shoot apex to trigger flowering. This scenario would explain why the *tsf* mutation does not cause a flowering time phenotype in itself, but does cause a flowering time phenotype in the absence of *FT* function.

Nevertheless, it remains an interesting question why TSF has a low ability to move. The finding that *TSF* misexpression from the *FT* promoter and *35S::TSF* cotyledon caused early flowering suggested that TSF can move from the leaf, whereas TSF movement is restricted from the rootstock. Thus, it seems likely that TSF movement is tissue-dependent (Kragler, 2013). In contrast, FT appears to move from the rootstock and the leaf. It is thus tempting to speculate that a component (or components) that is required to move TSF from the hypocotyl or rootstock may be absent in the hypocotyl or rootstock, but present in the leaf, although the possibility cannot be excluded that the failure of transport fluxes from the rootstock to the SAM of scion plants may have inhibit the movement of TSF in the grafting experiments. An alternative possibility is that although FT might travel in both phloem and xylem, TSF might travel only in the phloem. Thus, TSF may have moved inefficiently from the rootstock to the scion in these grafting experiments.

In summary, this study provides experimental data demonstrating that FT has a greater ability to move than TSF. However, replacing Region II of TSF with the FT sequence renders TSF graft-transmissible. The findings also show that *TSF* misexpression from the *FT* promoter and *35S::TSF* cotyledon accelerates flowering. Further investigation to identify the important residue(s) responsible for the difference in mobility of FT and TSF and identification of the molecule(s) that confer(s) the difference in mobility on FT and TSF will shed light on the molecular mechanism of floral induction by FT and TSF.

## Supplementary data

Supplementary data are available at *JXB* online.

Table S1. Oligonucleotide sequences used in this study.

Figure S1. TSF is highly expressed in the hypocotyl.

Figure S2. Butt-grafting strategy used in this study.

Figure S3. Flowering phenotype of scion plants grafted to ft and ft tsf rootstock plants.

Figure S4. Characterization of *35S::TSF* plants used in this study.

Figure S5. Expression of FT:T7 and TSF:T7 in the donor rootstock.

Figure S6. Sequence of FT/TSF chimeric proteins.

Figure S7. Flowering times of *ft tsf* scion plants grafted to independent lines of *FTF* and *TFT*-overexpressing plants.

Supplementary Data
