# Supplementary material for: FLOWERING LOCUS T has higher protein mobility than TWIN SISTER OF FT
Source: J Exp Bot. 2015 Jul 2;66(20):6109–17. doi: 10.1093/jxb/erv326 (PMC4588878; doi:10.1093/jxb/erv326)
Supplement: Supplementary Data [file supp_66_20_6109__index.html]

FLOWERING LOCUS T has higher protein mobility than TWIN SISTER OF FT — FLOWERING LOCUS T has higher protein mobility than TWIN SISTER OF FT — Supplementary Data 

# FLOWERING LOCUS T has higher protein mobility than TWIN SISTER OF FT

## Supplementary Data

Data files

- Supplementary Data - Supplementary Data
