# Supplementary material for: FLOWERING LOCUS T has higher protein mobility than TWIN SISTER OF FT
Source: J Exp Bot. 2015 Jul 2;66(20):6109–17. doi: 10.1093/jxb/erv326 (PMC4588878; doi:10.1093/jxb/erv326)
Supplement: Supplementary Data [file supp_erv326_jexbot148429_file001.pdf]

TITLE

FLOWERING LOCUS T (FT) has higher protein mobility than TWIN SISTER OF FT (TSF)

AUTHORS:

Suhyun Jin, Hye Seung Jung, Kyung Sook Chung, Jeong Hwan Lee, and Ji Hoon Ahn

CORRESPONDING AUTHOR:

Ji Hoon Ahn (J.H.A.)

Phone: 82-2-3290-3451; Fax: 82-2-927-9028; Email: jahn@korea.ac.kr

E-MAIL ADDRESS: letgo1003@korea.ac.kr (S. Jin)
dybin@korea.ac.kr (H.S. Jung)
kschung@korea.ac.kr (K.S. Chung)
1hwan3ee@korea.ac.kr (J.H. Lee)
jahn@korea.ac.kr (J.H. Ahn)

Supplementary Table

Table S1. Oligonucleotide sequences used in this study

Primer	Gene	Oligonucleotide sequence (5’ -> 3’)	Purpose
JH6350	*FT*	CTGGAACAACCTTTGGCAAT	qPCR
JH6351	*FT*	AGCCACTCTCCCTCTGACAA	qPCR
JH6488	*FT*	AGGCCTTCTCAGGTTCAAAACAAGC	qPCR
JH6489	*FT*	TGCCAAAGGTTGTTCCAGTTGTAGC	qPCR
JH6505	*PP2A*	GCGGTTGTGGAGAACATGATACG	qPCR
JH6506	*PP2A*	GAACCAAACACAATTCGTTGCTG	qPCR
JH6610	*TSF*	TATCTAGACACCAGCTGGTCCTGCATATCTG	Promoter cloning
JH6611	*TSF*	ACACCCGGGATTTATCTTGGATCTCAAGTATCTCAATC	Promoter cloning
JH7089	*FT*	TCCCCCGGGATGTCTATAAATATA	CDS amplification
JH7090	*FT*	TCCCTCGAGCTAAAGTCTTCTTCCT	CDS amplification
JH7306	*TSF*	CACCACTGGAAATGCCTTTGGC	qPCR
JH7308	*TSF*	CGAGTTGCCGGAACAATACCAAC	qPCR
JH8950	*FT*	CCATGATTACGAATTCGATTACCTCCCAGCACCAAAGACA	Promoter cloning
JH9120	*FT*	CATCCCCGGGTACCGAGCTCCTTTGATCTTGAACAAACAGGTGGTT	Promoter cloning
JH9124		CAAAGGAGCTCGGTACCCATGTACCCATACGATGTTCCAGAT	HA:FT/TSF:T7 amplification
JH9125		CGACTCTAGAGGATCCCCCTATCCCATTTGTTGTCCTCCAGTCA	HA:FT/TSF:T7 amplification

Underlined letters denote synthetic restriction enzyme sites.

Supplementary Figure legends

Figure S1. *TSF* is highly expressed in the hypocotyl.
(A) Histochemical staining of *gTSF:GUS* in wild-type plants under LD conditions. Note that *gTSF:GUS* staining was mainly seen in the hypocotyl during the vegetative phase. Expression of *pFT::GUS* is shown for comparison. Upregulation of *APETALA1* (*AP1*) began to increase after D9 under our growth conditions (Yoo *et al.*, 2013b), indicating that seedlings after D10 were in the reproductive phase under our growth conditions. During the reproductive phase (D20), strong *TSF* expression was detected in the petiole of the true leaf.
(B) *In silico* expression profiling of *TSF* from publicly available microarray data showing high *TSF* expression (highlighted in a grey box) in the hypocotyl (Zimmermann et al., 2004).

Figure S2. Butt-grafting strategy used in this study.
Note that the scion was prepared to contain minimal hypocotyl tissue. The cotyledon in scion plants was removed before grafting union. H: hypocotyl; R: root

Figure S3. Flowering phenotype of scion plants grafted to *ft* and *ft tsf* rootstock plants. Scale bar = 1 cm

Figure S4. Characterization of *35S::TSF* plants used in this study.
(A) qPCR confirmation of strong expression of *TSF* in *35S::TSF* rootstock plants that we used.
(B) Strong early flowering of *35S::TSF* plants used for butt-grafting experiments. Scale bar = 1 cm

Figure S5. Expression of FT:T7 and TSF:T7 in the donor rootstock.
FT:T7 and TSF:T7 protein expression was detected by western blot analysis using anti-T7 antibody (top). Rubisco was used as a loading control (bottom). Total protein extracted from wild-type Col-0 plants was used for a negative control (NC).

Figure S6. Sequence of FT/TSF chimeric proteins
(A) Amino acid sequence alignment of FT and TSF using ClustalW (Larkin et al., 2007). Asterisks indicate identical residues. For conserved changes, a dot (.) indicates conservation between groups with weakly similar properties (scoring ≤ 0.5 in the Gonnet PAM 250 matrix) and a colon (:) indicates conservation between groups with strongly similar properties (scoring > 0.5 in the Gonnet PAM 250 matrix). Three regions selected for generating chimeric constructs are indicated.
(B) Comparison of the three regions that were used to generate chimeric constructs.

Figure S7. Flowering times of *ft tsf* scion plants grafted to independent lines of *FTF*- and *TFT*-overexpressing plants.
Distribution of flowering time is presented as a heatmap. The structure of each chimeric gene is shown next to the name of each construct. Sequences of *FT* and *TSF* are shown as gray and open boxes, respectively. n = number of plants measured. *F*: the region that was originated from *FT*; *T*: the region that was originated from *TSF*.

Supplementary Figures

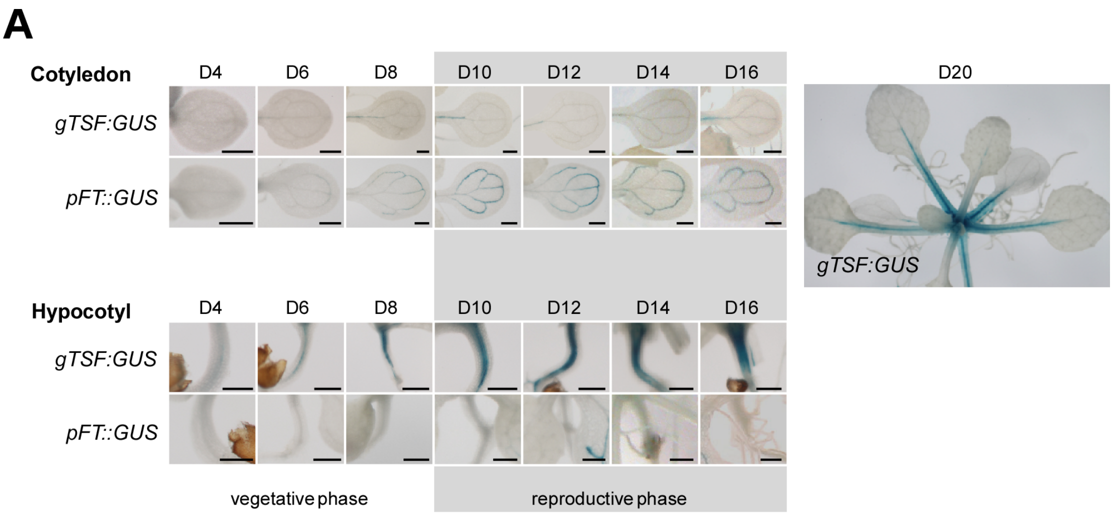

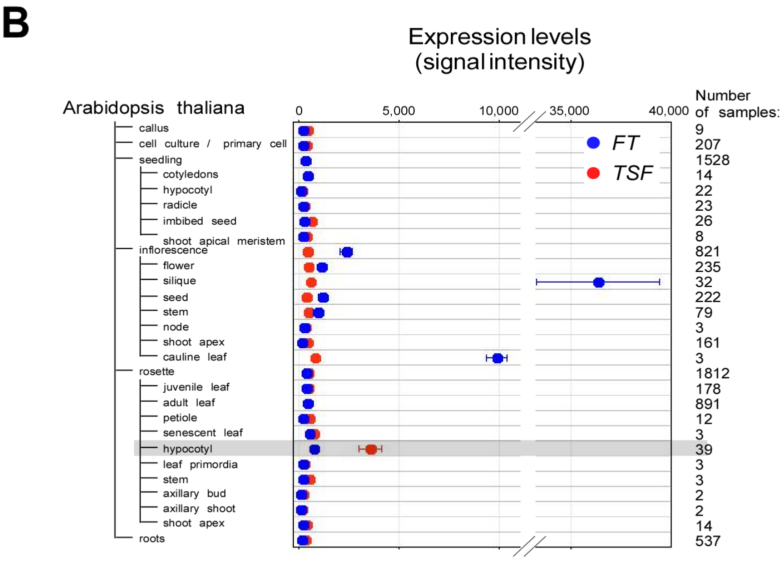

Figure S1

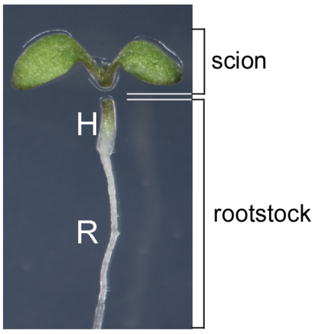

Figure S2

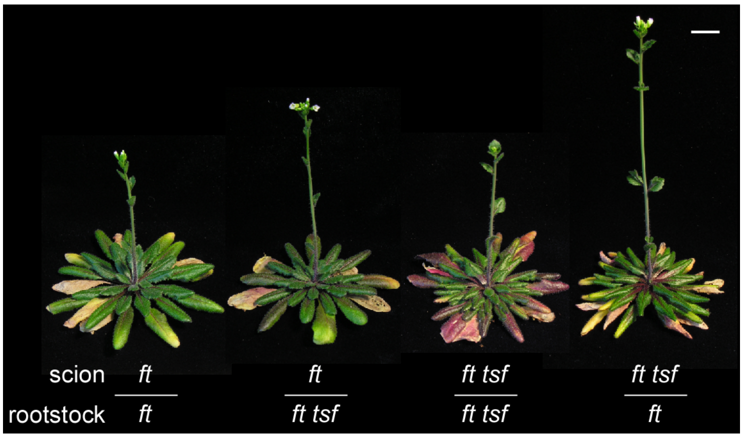

Figure S3

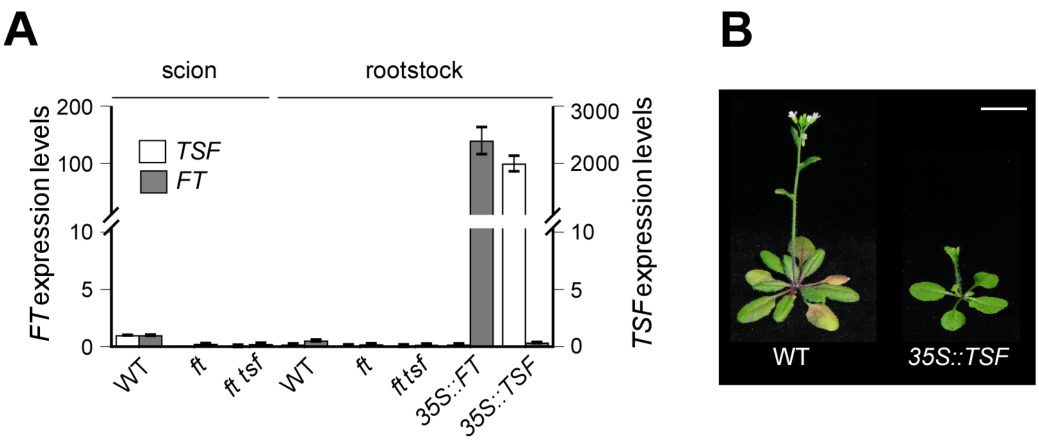
A
scion
rootstock
TSF
FT
FT expression levels
TSF expression levels
200
100
10
5
0
3000
2000
WT
ft
ft tsf
35S::FT
35S::TSF
B
WT
35S::TSF

Figure S4

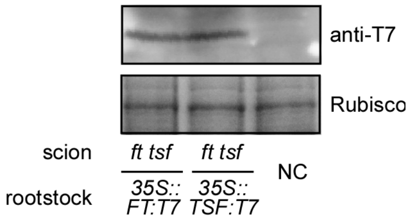
anti-T7
Rubisco
scion
ft tsf
ft tsf
NC
rootstock
35S::
FT:T7
35S::
TSF:T7

Figure S5

A

```
       ----------------- I ----------------->||<------------------ II -------------------
FT     MSINIRDPLIVSRVVGDVLDPFNRSITLKVTYGQREVTNGLDLRPSQVQNKPRVEIGGED 60
TSF    MSLSRRDPLVVGSVVGDVLDPFTRLVSLKVTYGHREVTNGLDLRPSQVLNKPIVEIGGDD 60
       **:. ****:*. *********.* ::******:************** *** *****:*
       ------------------------------------->||<------- III ---------
FT     LRNFYTLVMVDPDVPSPSNPHLREYLHWLVTDIPATTGTTFGNEIVCYENPSPTAGIHRV 120
TSF    FRNFYTLVMVDPDVPSPSNPHQREYLHWLVTDIPATTGNAFGNEVVCYESPRPPSGIHRI 120
       :******************** ****************.:****:****.* *.:****:
       -------------------------------------------------------
FT     VFILFRQLGRQTVYAPGWRQNFNTREFAEIYNLGLPVAAVFYNCQRESGCGGRRL 175
TSF    VLVLFRQLGRQTVYAPGWRQQFNTREFAEIYNLGLPVAASYFNCQRENGCGGRRT 175
       *::*****************:****************** ::*****.******
```

B

	total residues	different residues (%)	nonconserved change	conserved change .	conserved change :
Region I	27	10 (37)	3	3	4
Region II	71	6 (8)	3	-	3
Region III	77	16 (20)	3	4	9

Figure S6

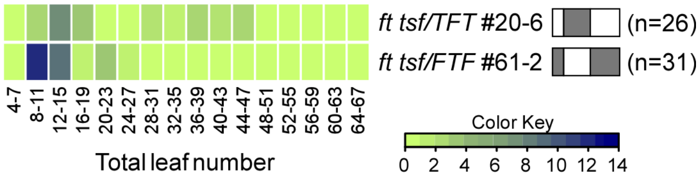
ft tsf/TFT #20-6
(n=26)
ft tsf/FTF #61-2
(n=31)
4-7
8-11
12-15
16-19
20-23
24-27
28-31
32-35
36-39
40-43
44-47
48-51
52-55
56-59
60-63
64-67
Total leaf number
Color Key
0 2 4 6 8 10 12 14

Figure S7
